# Jasmonates elicit different sets of stilbenes in *Vitis vinifera* cv. Negramaro cell cultures

**DOI:** 10.1186/s40064-015-0831-z

**Published:** 2015-02-01

**Authors:** Marco Taurino, Ilaria Ingrosso, Leone D’amico, Stefania De Domenico, Isabella Nicoletti, Danilo Corradini, Angelo Santino, Giovanna Giovinazzo

**Affiliations:** Institute of Food Production Sciences, C.N.R. Unit of Lecce, via Monteroni, 73100 Lecce, Italy; Institute of Chemical Methodologies, CNR, Area della Ricerca di Roma 1, via Salaria km 29,300, Monterotondo Stazione, Rome, Italy

**Keywords:** *Vitis vinifera* cv. Negramaro, Methyl jasmonate, Jasmonic acid, 12-oxo-phytodienoic, Coronatine, Chitosan, Viniferins

## Abstract

The plant phenol *trans-*resveratrol, which is mainly found in grape, displays a wide range of biological effects. A cell suspension culture was developed from calli of grape leaves of *Vitis vinifera* cv. Negramaro in order to study the bioproduction of resveratrol. The effects of a number of secondary plant metabolism elicitors, namely chitosan, methyl jasmonate, jasmonic acid, coronatine, and 12-oxo-phytodienoic acid, were tested on this cell suspension culture. The identification and quantification of stilbenes was achieved with high performance liquid chromatography, with both spectrophotometric and mass spectrometric detection. Of the tested elicitors, methyl jasmonate was the most effective in inducing the biosynthesis of approximately 4 mg g^−1^ dry weight (about 60 mg L^−1^) of resveratrol. Conversely, 12-oxo-phytodienoic acid, jasmonic acid, and coronatine were able to trigger the synthesis of approximately 20 mg g^−1^ dry weight (200–210 mg L^−1^) of viniferins. Taken together, our results show for the first time different modulatory effects of closely-related jasmonates on stilbene biosynthesis.

**Electronic supplementary material:**

The online version of this article (doi:10.1186/s40064-015-0831-z) contains supplementary material, which is available to authorized users.

## Introduction

Phytoalexins from grapevine (*Vitis vinifera* L.) mainly belong to the stilbene family (Ahuja et al. 2012; Jeandet et al. 2010). *trans*-Resveratrol (*t-*R), resveratrol glucosides, as *trans*-piceid (*t-*P), methylated derivatives, and oligomers (as α-viniferin and ε-viniferin) represent a class of defence compounds whose synthesis is induced upon several (a)biotic stresses (Jeandet et al. 2002; Alonso-Villaverde et al. 2011; Dubrovina et al. 2010; Kiselev 2011).

Many studies have reported that daily consumption of *t*-R is associated with beneficial effects and protection against several human pathologies including coronary heart diseases, neurodegenerative disease, and different types of cancers (Flamini et al. 2013; Schrauwen and Timmers 2014). Among the biotechnological approaches used to produce *t*-R the synthesis of resveratrol, there are a number of examples of genetically modified plants in which the production of this phytochemical was induced by genetic insertion of the stilbene synthase gene, which encoded the enzyme responsible for the biosynthesis step (Giovinazzo et al. 2012; Jeandet et al. 2012; Jeandet et al. 2013).

Interesting results can also arise from the potential of genetically modified microorganisms as an alternative mechanism for producing resveratrol. Metabolic engineering to tailor *Escherichia coli* and yeast with structural genes of resveratrol pathway, missing in these organisms, has been successfully reported (Watts et al. 2006; Katsuyama et al. 2007; Donnez et al. 2009; Shin et al. 2012).

Another potential approach is mediated by plant cell cultures in which the production of stilbenes is induced by the addition of specific elicitors to the culture medium. The amount of *t*-R produced in response to elicitors widely fluctuates according to plant species, elicitor type, and culture conditions (Donnez et al. 2009). Furthermore, grape cell suspension cultures represent a reliable model system for basic research on plant defence mechanisms and *in vitro* production of phenols (Donnez et al. 2009). A number of studies using grape cell suspension cultures and microorganisms have reported improved production of resveratrol.

In grape cell cultures, sucrose, in combination with elicitors, methyl jasmonate (MeJA) and cyclodextrins (CDs) or methyl jasmonate and sodium orthovanadate, up-regulated the expression of defence genes and enhanced the accumulation of stilbenes (Belchí-Navarro et al. 2012; Belhadj et al. 2008; Lijavetzky et al. 2008; Tassoni et al. 2005). Furthermore, the extracellular accumulation of *t*-R was also reported when modified CDs (heptakis (2,6-di-*O*-methyl)-β-cyclodextrin and methyl-β-cyclodextrin) were added to grapevine cell cultures (Morales et al. 1998; Ahuja et al. 2012; Lijavetzky et al. 2008). Transcriptomic analyses of grapevine cells in response to methyl-β-cyclodextrin confirmed the induction of a specific set of genes belonging to the phenylpropanoid metabolism (Zamboni et al. 2009).

Taken together, these results indicate that efforts to induce stilbene accumulation should include a combination of different approaches and identification of new elicitors.

The aim of the present study was to investigate the ability of biotic elicitors to increase stilbene production, particularly *t*-R and viniferins, in cell suspension cultures of *V. vinifera* cv. Negramaro. The effects of well-known elicitors, i.e. MeJA, jasmonate (JA), and chitosan (CHI), were compared with those of 12-oxo-phytodienoic acid (OPDA), a biologically active intermediate of the jasmonate biosynthesis pathway, and coronatine (COR), a phytotoxin produced by the *Pseudomonas syringae* species (Bender et al. 1999), which is able to mimic the biologically active form of JA, jasmonoyl-isoleucine (JA-Ile) (Ichihara et al. 1977).

OPDA has been linked to insect defence and might act as an antifungal compound (Nilsson et al. 2012). Indeed, in addition to its obvious role as the precursor of JA, OPDA seems to play an independent role in mediating resistance to microorganisms and pests (Nilsson et al. 2012). Regarding COR, it was reported to induce a wide array of effects in plants such as the strong activation of defence (Lauchli and Boland 2003).

The results reported here indicate that jasmonates are able to trigger different sets of stilbenes in cv. Negramaro cell culture.

## Materials and methods

### Establishment of callus cultures

Callus tissues were obtained from leaves of 10-year-old *V. vinifera* L. cv. Negramaro plants at the phenological stage of pea-sized berries. The leaf tissues were surface-sterilised with 70% (v/v) ethanol for 2 min and 1% (v/v) sodium hypochlorite for 10 min, and finally rinsed three times with sterile distilled water. The leaves were then cut into 1-cm pieces and placed onto a solid G5V culture medium containing Gamborg B5 salts and vitamins (Gamborg et al. 1968), 30 g L^−1^ sucrose, 250 mg L^−1^ hydrolysed casein, 0.5 g L^−1^ polyvinylpyrrolidone (PVP-40), 0.2 mg L^−1^ kinetin, 0.1 mg L^−1^ α-naphthaleneacetic acid, and 7 g L^−1^ agar, adjusted to pH 5.6, and maintained at 25°C in the dark. After 3–4 weeks, the different callus tissues were separated from the explants and maintained. Grapevine calli were maintained at 25°C in the dark and sub-cultivated on a solid growth medium every month.

### Establishment of cell suspension cultures

*V. vinifera* cv. Negramaro cell suspension cultures were initiated by inoculating one-year-old friable callus pieces (about 1 g fresh weight [FW]) in 250 mL Erlenmeyer flasks containing 50 mL of liquid G5V medium adjusted to pH 6.0. The flasks were kept in a rotary shaker (110 rpm) at 25°C in the dark. Negramaro cell suspension cultures were routinely maintained by periodical subculture every 10–14 days by dilution with one volume of growth medium and then distribution into two flasks.

### Microscopic observation of the cells

Cell viability was assessed by incubating the cells for 1 min in a fresh medium containing fluorescein diacetate (FDA) at a final concentration of 4 μg mL^−1^. Images of the cells were taken on a confocal laser microscope (LSM Pascal Zeiss). One–micrometre-thick sections were imaged with an excitation wavelength of 488 nm. FDA fluorescence was detected with a FITC filter (520 nm).

### Elicitor treatments

Elicitation experiments were performed in triplicate using 10- to 14-day-old Negramaro cell suspensions at the moment of subculture. At this stage of cell development, 10 g FW of cells were transferred into 500 mL flasks and suspended in 50 mL of fresh growth medium (20% concentration). At day 0, cell cultures were supplied with 50, 100, and 150 μM MeJA (dissolved in 100% ethanol), 10 μM COR (dissolved in 100% ethanol), and 100 μM OPDA (dissolved in 100% ethanol). Furthermore, 100 μM JA (dissolved in 100% dimethyl formamide) was also used, in combination with 100 μg mL^−1^ CHI (dissolved in 0.1% acetic acid). Control cultures (in triplicate for each elicitor) were obtained by adding appropriated solvents to the GV5 medium. Untreated cells were also utilised as control in all experiments. Elicitor-treated and control cells were harvested during growth, centrifuged, rapidly washed, weighed, frozen in liquid nitrogen, and stored at −80°C or dried and stored at 5°C for further analyses.

### Statistics

Statistical analysis was performed with GraphPad Prism. All data are the mean of three measurements ± standard deviation (SD). Multifactorial analysis of variance (ANOVA) followed by the Tukey multiple comparison test was carried out on different growth conditions (dark/light), or the effect of different elicitors on stilbenes production at all the time points here considered. A p-value of <0.05 was taken to indicate significant differences.

### Identification and quantification of stilbenes

Aliquots of cell cultures (10 mL, cell weight was determined for each treatment) were collected after elicitor or solvent treatment from control and treated samples. Cells and medium were separated by centrifugation at 1500 g for 5 min at room temperature. Pellets were ground to a fine powder in liquid nitrogen and lyophilised. Medium was frozen in liquid nitrogen and lyophilised. Dried cells (100 mg) and dried medium (100 mg) were treated with 80% methanol (10 mL) and put on a rotary shaker (110 rpm) at 25°C in the dark for 12–13 h. The hydro-alcoholic extracts were dried under vacuum (at about 30°C), dissolved in the extractive mixture (200 μL), then filtered through a 0.22 μm membrane (Whatman, New Jersey, USA) and 20 μL analysed with reversed-phase high-performance liquid chromatography (RP-HPLC).

The instrument used for RP-HPLC analysis was a Model LCMS-2010 liquid chromatograph equipped with a Model SPD-M10Avp photodiode array (PDA) detector, which was hyphenated with a Model 2010 single quadrupole mass spectrometer by an electrospray ionisation (ESI) source, all from Shimadzu (Milan, Italy). System control and data processing were carried out with the Shimadzu LCMS solution software. A M8125 semi-micro injection valve (Rheodyne, Cotati, CA, USA) with a 5-μl sample loop was used for sample injection and the chromatographic separations were carried out on a reversed-phase Polaris C18A column (150 × 2.0 mm ID, 5 μm) equipped with a C18 (30 × 2 mm ID, 5 μm) guard cartridge column (Varian Inc., Lake Forest, CA, USA), both thermostated at 30°C ± 1°C by a DBS (Vigonza, Padua, Italy) Model PCO 200 column oven.

Samples were eluted by a multi-segment gradient of increasing concentration of acetonitrile in water, containing 1.0% (v/v) formic acid, according to the following programme: 25 min linear gradient from 18 to 40% (v/v) acetonitrile, followed by a 3 min steeper linear gradient to 58% (v/v) acetonitrile, 1 min linear gradient to 60% (v/v) acetonitrile and 2 min isocratic elution with 60% (v/v) acetonitrile (Nicoletti et al. 2007). Then the eluent composition was brought to the initial condition in 1.0 min, and the column equilibrated for 15.0 min before the next injection. A flow rate of 0.2 ml min^−1^ was used for both sample elution and column equilibration. The MS detection and mass spectra acquisition were carried out in negative ionisation mode with the ESI interface at the following conditions: temperature of block heater, 200°C; temperature of the curved desolvation line (CDL), 225°C; probe voltage, −3.5 V; CDL voltage, 25 V; Q-array voltages, 0, −15, and −60 V; and Q-array RF, 150 V, using nitrogen as the nebulising gas at a flow rate of 4.5 L/min.

The individual stilbenes were identified on the basis of their mass data, obtained with ESI-MS in the negative single ion monitoring (SIM) mode, and ultraviolet UV spectra, acquired with a PDA detector in the wavelength range between 210 and 400 nm, which were compared either with those reported in the literature (Vergara et al. 2012; Donnez et al. 2011; Kong et al. 2011) or with authentic standards. The content of *t*-R and of *trans*-piceid (*t*-P) was determined on the basis of calibration graphs obtained with solutions of authentic standards at six different concentration levels (0, 15, 30, 60, 120, 250 μg mL^−1^), whereas the amount of viniferins was expressed in terms of *t*-R, using the calibration graph constructed for this stilbene. Authentic standards of *t*-R and of *t*-P were purchased from Sigma-Aldrich (Milan, Italy) and Polyphenols Laboratories AS (Sandnes, Norway), respectively, whereas *cis-* and *trans*-ε-viniferins were a kind gift from Dr V. Flors (University of Castellon, Spain).

## Results and discussion

### Establishment of *V. vinifera* Negramaro grape cell cultures to optimise stilbene biosynthesis

With the purpose of optimising a grape cell culture system of the *V. vinifera* cv. Negramaro grape, calli were obtained from leaflet explants grown on agarised G5V medium (see Materials and methods). At day 30 of culture, 90% of the explants produced calli. Two different calli lines, named A and B, were obtained. The young calli, after 3–4 weeks of sub-cultivation in the dark, were exposed to light in order to induce stilbene biosynthesis. In this condition, the line named as A developed red pigmentation, suggesting that the flavonoid pathway could be completely induced by light. In the same callus line, the red pigmentation vanished and the colour of the culture turned to green, as a consequence of chlorophyll synthesis, when it was transferred to a liquid medium. However, cell proliferation was low in liquid culture conditions.

**Figure 1:**
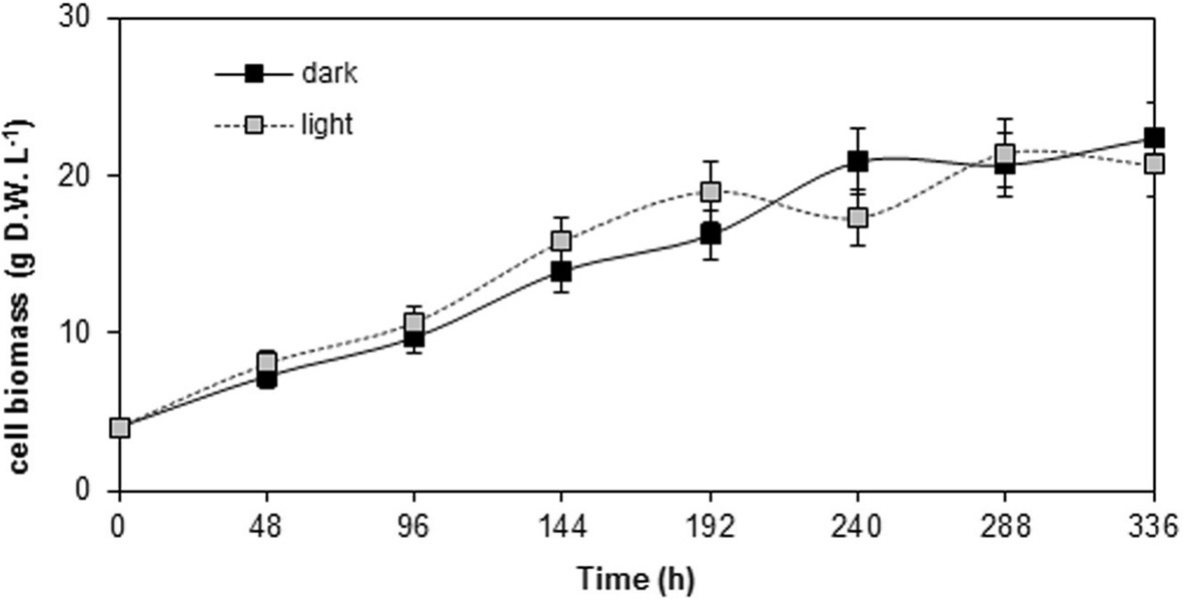
**Growth curves of** ***V. vinifera*** **cv Negramaro cell suspension cultures maintained under light and in the dark.** Growth was determined by sampling aliquots (10 ml) of the cell suspension at different time points from 0 to 336 h. Error bars correspond to the standard deviation from three extractions.

By contrast, the line named as B did not show any red pigmentation when transferred to light. Furthermore, its rapid growth and friable status suggested that it might be suitable for being transferred to a liquid medium. The growth of this cell line was monitored by measuring its dry weight up to 14 days of cultivation when exposed to white light or maintained in darkness. Both the light-exposed and dark-maintained cell cultures displayed an exponential growth phase between day 4 and 10 (96–240 hours), followed by a stationary phase until at least day 14 (336 hours) (Figure 1). On the basis of the results relating to the growth rate and other physiological features, line B was chosen for subsequent elicitation experiments.

### Identification and quantification of stilbenes with RP-HPLC-PDA-ESI-MS

**Figure 2:**
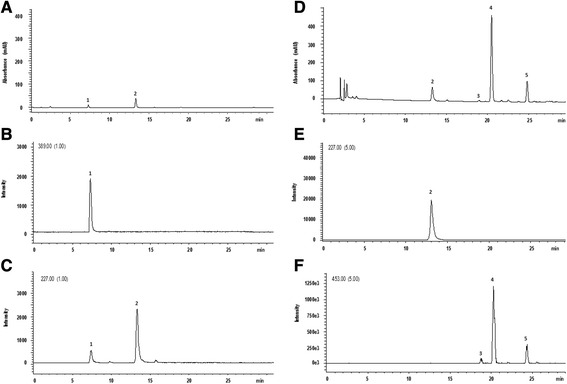
**Identification and quantification of stilbenes in** ***V. vinifera*** **cv Negramaro.** Representative chromatograms of the sample extracted from non-elicited cells (control), analyzed by RP-HPLC and detected by PDA at 306 nm **(A)** and by ESI-MS in negative single ion monitoring (SIM) mode at *m/z* 389 **(B)** and *m/z* 227 **(C)**. RP-HPLC-PDA-ESI-MS conditions as in the Experimental section. Identification of peaks (see text): 1, *trans*-piceid; 2, *trans*-resveratrol. Representative chromatograms of the sample extracted from cells treated with OPDA, analyzed by RP-HPLC and detected by PDA at 306 nm **(D)** and by ESI-MS in negative single ion monitoring (SIM) mode at *m/z* 227 **(E)** and *m/z* 453 **(F)**. RP-HPLC-PDA-ESI-MS conditions as in the Experimental section. Identification of peaks (see text): 2, *trans*-resveratrol; 3, *cis*-ε-viniferin; 4, *trans*-ε-viniferin; 5, *trans*-δ-viniferin.

Stilbenes were extracted from control and elicited cell cultures and analysed with RP-HPLC with PDA and ESI-MS detection, as described in the Materials and methods section. Figure 2 show representative chromatograms of the samples extracted from dried cells, control and cell cultures treated with OPDA, respectively. Analogous chromatograms, with deviations of retention times of the detected peaks within 0.8% (RSD), were also obtained for the samples extracted from cells elicited with all the elicitors used in this study. In Figure 2, panel A displays the chromatograms recorded by the PDA detector at 306 nm whereas panels B and C show the extracted-ion chromatograms of the ion current at *m/z* values corresponding to the ions of the individual investigated compounds, detected by ESI-MS in negative ionization mode.

The identity of peaks 1–5 was established on the basis of retention times and UV spectra of authentic standards, recorded in the 210–400 wavelength range, and further confirmed by ESI-MS detection in the single ion monitoring (SIM) mode at *m/z* value corresponding to the main ion of the searched compounds, which were detected in correspondence with the peaks displayed by the PDA chromatogram. Under the selected experimental conditions, fragmentation of the investigated compounds was very limited and, therefore, the main observed ion in the ESI-MS spectra acquired in negative ionisation mode coincided with the deprotonated molecular ion [M-H]^−^. An exception to this general trend was observed for peak 1, which was identified as the monoglycosylated stilbene *t-*P (MW 390), which underwent fragmentation giving rise to a product ion corresponding to its aglycone *t*-R. Panel B of Figure 2 clearly shows that acquiring the extracted-ion chromatogram of the ion current at *m/z* 389, corresponding to the [M-H]^−^ ion, evidenced the presence of a peak at the retention time corresponding to peak 1. The identity of this peak was further investigated by acquiring the extracted-ion chromatogram of the ion current at *m/z* 227, corresponding to the [M-H-162]^−^ ion resulting from the loss of a 162 mass fragment, equivalent to the hexose group of the glycosylated *t-*R, which also appeared in correspondence with peak 1 (see panel C of Figure 2).

Peak 2 was detected in both samples extracted from control and cell cultures treated with elicitors. The identity of this peak, whose retention time and UV spectra resembled those of standard *t-*R analysed under identical conditions, was confirmed by acquiring the extracted-ion chromatogram of the ion current at *m/z* 227, corresponding to the [M-H]^−^ ion of the above stilbene (see panel E of Figure 2).

The retention times of peak 3 and 4, eluting at 18.8 and 20.3 min, respectively, were within 0.8% of those of *cis*- and *trans*-ε-viniferins, respectively, analysed as authentic standards. These peaks, together with peak 5, eluting at 24.5 min, were also detected by ESI-MS in SIM detection mode, acquiring the ion current at *m/z* 453, corresponding to the [M-H]^−^ ions of viniferins (see Figure 2 panel F). A possible attribution of the identity of each of these peaks was performed on the basis of the analysis of UV spectra, acquired by the PDA detector in the wavelength range between 210 and 400 nm. Accordingly, the most abundant peak eluting at 20.3 min (peak 4) was identified as *trans*-ε-viniferin on the basis of the characteristic UV absorption maximum measured at 322 nm (Vergara et al. 2012), whereas the two smaller peaks detected by ESI-MS in SIM mode at 18.8 (peak 3) and 24.5 min (peak 5) were attributed to *cis*-ε-viniferin and *trans*-δ-viniferin, respectively, on the basis of their characteristic UV absorption maxima at 282 nm and 309 nm, respectively (Donnez et al. 2011, Kong et al. 2011). The identified viniferins were quantified on the basis of the corresponding peak areas using the calibration graph constructed with *t-*R as the external standard (Krisa et al. 1999).

### Basal levels of stilbenes in dark-maintained and light-exposed cell cultures

**Figure 3:**
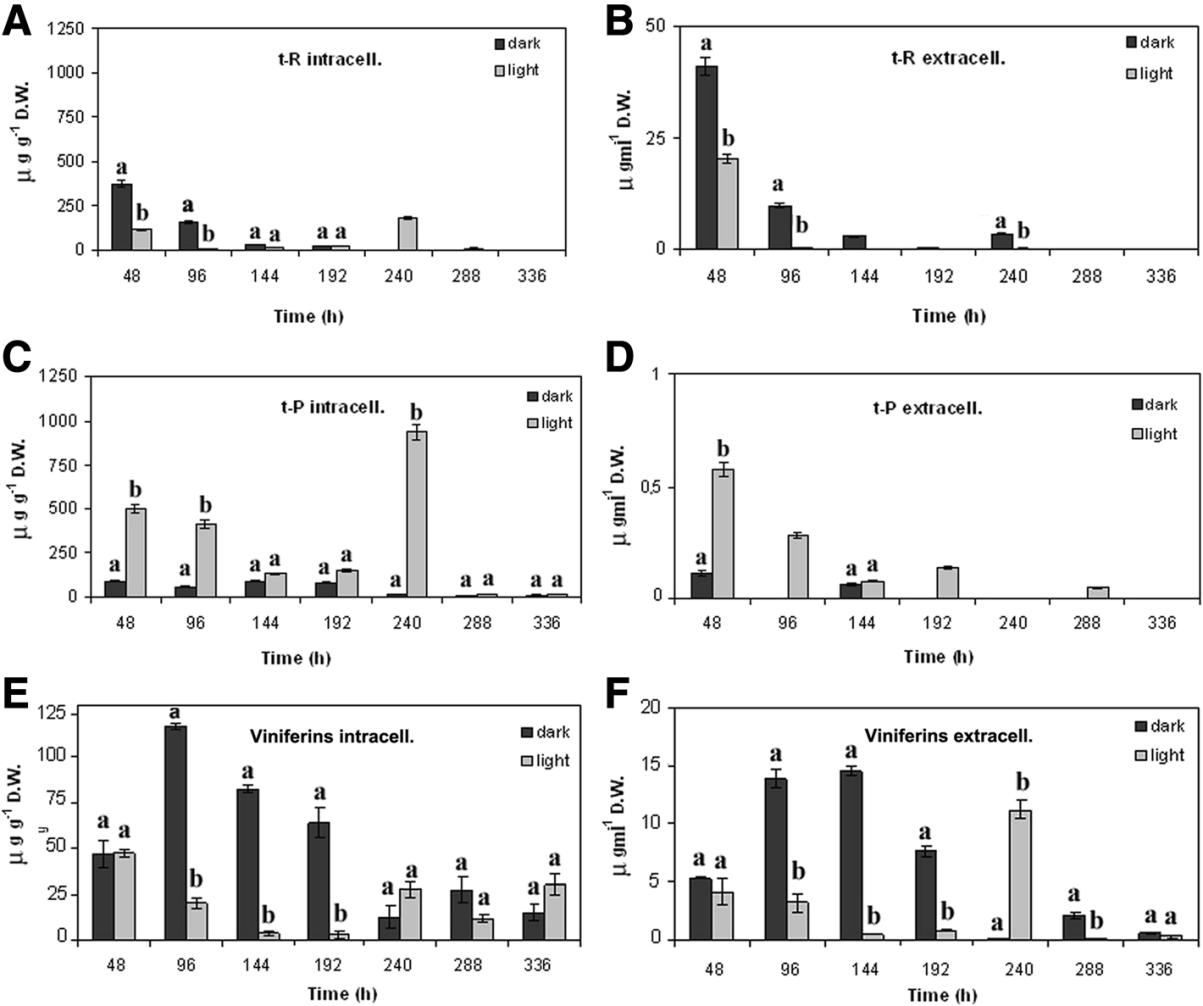
**Stilbene production in** ***V. vinifera*** **cv Negramaro non treated cell suspension cultures maintained under light and in the dark.** Quantification of intracellular **(A, C, and E)** and extracellular **(B, D, and F)** levels of *trans*-resveratrol (*t*-R), *trans*-piceid (*t*-P), and viniferins was carried out by RP-HPLC using chemically synthesised compounds as external standards. Within any given quantification, bars with different letters indicate significant differences between different conditions (p < 0.05, ANOVA test).

In order to induce the stilbene biosynthesis pathway in the Negramaro cell line, the callus lines were cultivated for 3–4 weeks under light. Stilbene levels were quantified by RP-HPLC in dark-maintained and light-exposed cell lines. In both conditions, stilbene levels were low. However, the dark-maintained cell cultures showed the higher production of *t-*R in comparison with the light-exposed ones, with a maximum yield of about 300 μg g^−1^ of DW (corresponding to 2.4 mg L^−1^) recorded at 48 h (Figure 3A). Unlike *t-*R, *t-P* accumulated mainly in the light-exposed calli, with a peak at 240 h, with about 1 mg g^−1^ of DW (corresponding to 24 mg L^−1^). Finally, low levels of viniferins were also detected in our experimental conditions. Similarly to *t-*R, viniferins accumulated mainly in the dark-maintained cell cultures and reached the highest concentration at 96 h, with about 120 μg g^−1^ of DW (corresponding to 1.4 mg L^−1^). It is worth noting that in our experimental conditions, the levels of stilbenes secreted in the medium were always very low in comparison with the levels accumulated intracellularly (Figure 3B-F). On the basis of the recorded levels of resveratrol and viniferins, and the observation that the light-exposed cell cultures showed a greater tendency to aggregate and form greenish clusters, we decided to carry out elicitation experiments using the dark-maintained cell lines.

### Effects of MeJA and CHI on the production of resveratrol in *V. vinifera* cv. Negramaro cell suspension

MeJA and CHI are well-known compounds widely used in the elicitation of secondary metabolites in grape cell cultures (Ferri et al. 2011; Santamaria et al. 2011; Vuong et al. 2014). In preliminary experiments, we tested the effects of different concentrations of MeJA on cell viability and stilbene biosynthesis (Additional file 1: Figure S1 and Additional file 2: Figure S2). Firstly, FDA staining was used to evaluate cell viability. The results (shown in Additional file 1: Figure S1C, F, and I) indicated the presence of large aggregates of unstained cells after elicitation with 150 μM MeJA, thus pointing to the presence of unviable cells (about 70%). In line with this observation, the highest levels of *t*-R production, but not for *t*-P, were recorded with a MeJA concentration of 100 μM. At higher concentrations of MeJA, resveratrol levels were lower, likely as a consequence of the increased cell mortality.

**Figure 4:**
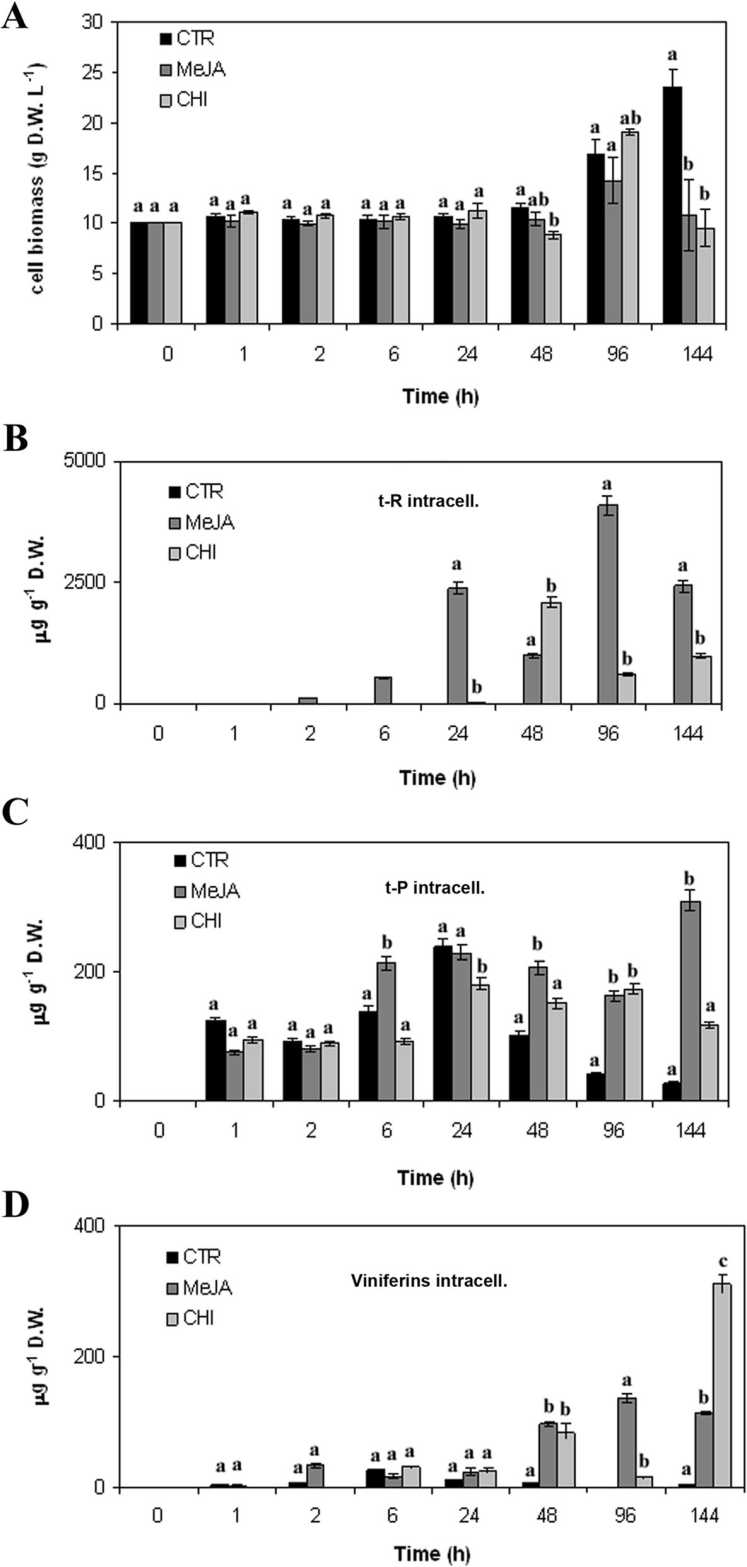
**Stilbene production in** ***V. vinifera*** **cv. Negramaro cell cultures after elicitation with MeJA and CHI.** Determination of cell biomass of *V. vinifera* cv Negramaro cultures elicited with 100 μM MeJA and 50 μg ml^−1^ CHI at different time points **(A)**. Quantification of *trans*-resveratrol (t-R, **B**), *trans*-piceid (*t*-P, **C**), and viniferins **(D)** produced by Negramaro cell suspension cultures treated with different elicitors. Quantification was carried out by RP-HPLC using chemically synthesised compounds as external standards. Each point is the average of three independent experiments. Within any given quantification, bars with different letters indicate significant differences between different treatments (p < 0.05, ANOVA test).

CHI was used at the same concentration as in elicitation experiments carried out on other grape cultivars (Santamaria et al. 2011). The effects of MeJA and CHI elicitation on *V. vinifera* cv. Negramaro cell growth were first monitored measuring cell weight (DW per L^−1^) from 1 to 144 h (Figure 4A). In our experimental conditions, treatment with these two elicitors had no evident inhibitory effect on the growth of grape cell cultures up to 96 h. Indeed, the weight of elicited and control grape cell cultures showed small variations up to 48 h and started to slightly increase at 96 h (Figure 4A). Afterwards, cell growth decreased in elicited cultures, whereas it exponentially increased in control samples. The cell growth decrease in elicited cultures was most likely due to the cytotoxic effect of the elicitors added to the medium (about 90% mortality, data not shown).

Since our purpose was to optimise stilbene accumulation, we followed the production of these phytochemicals for up to 6 days (144 h) after elicitation. All samples extracted from the elicited cultures at different time points were analysed with RP-HPLC-PDA-ESI-MS for the identification and quantification of stilbenes, according to the method described in the experimental section.

A number of studies using grape cell suspension cultures have reported an improved production of resveratrol, using chemically different elicitors. In *V. vinifera* cv. Chasselas × *V. berlandieri* cell suspension cultures, MeJA at a concentration of 200 μM induced up to 150 mg L^−1^ of resveratrol in a flask system and 209 mg L^−1^ in a 2 L-stirred bioreactor (Donnez et al. 2011). In our cell system, a rapid accumulation of *t*-R was recorded when the grape cell cultures were treated with MeJA, starting from 2 h (Figure 4B) and reaching its maximum value at 96 h, with about 4000 μg g^−1^ DW (corresponding to 56 mg L^−1^), and decreasing afterwards.

Conversely, the content of *t*-R after treatment with CHI, a derivative of chitin, was only slightly higher than that of control cultures, reaching the highest concentration after 48 h with about 2000 μg g^−1^ DW (corresponding to 16 mg L^−1^), and decreasing afterwards. In *V. vinifera* cv. Gamay Fréaux (Vuong et al. 2014), the addition of a lower concentration of CHI (from 0.5 to 25 μg mL^−1^ CHI) increased the level of intracellular resveratrol by around five-fold (about 5–6 mg L^−1^) in comparison with control, after 144 h (day 7). CHI (50 μg mL^−1^) was also found to increase the intracellular accumulation of monoglucosylated resveratrol from 3 to 10.5-fold in *V. vinifera* cv. Barbera cell cultures (Ferri et al. 2011), thus reaching a level of elicitation similar to those reported by Vuong et al. (2014). However, the different levels of intracellular *t*-R between *V. vinifera* cv Negramaro and cv Gamay Fréaux (Vuong et al. 2014), cell cultures, could be due to the higher concentration of CHI (100 μg mL^−1^) and different cultivar utilised in the present study. Regarding the elicitation of *t*-P, no significant differences were found up to 48 h. The highest levels were recorded in cell cultures elicited with MeJA for 144 h (Figure 4C), in which *t*-P reached a concentration of about 300 μg g^−1^ DW (corresponding to 3.3 mg L^−1^).

Finally, both MeJA and CHI were also able to elicit the production of low levels of viniferins, with the highest levels recorded in cell cultures elicited with CHI for 144 h (about 320 μg g^−1^, corresponding to 2.6 mg L^−1^; Figure 4D). Taken together, this first set of elicitation experiments indicated that either MeJA or CHI were mainly able to trigger the synthesis of *t*-R, since either *t*-P or viniferins were accumulated at a much lower level (less than ten-fold) than resveratrol. In our experimental condition the levels of stilbenes secreted in the medium were always very low in comparison with the amount accumulated intracellularly (data not shown).

### Effects of OPDA, JA, and COR on the growth of *V. vinifera* cv. Negramaro cell suspension cultures and on stilbene production

MeJA and JA are well-known elicitors that are widely used in biotechnological production of phytochemicals (Donnez et al. 2009). It is known that jasmonates are components of a network of a signal transduction pathways involved in plant defence response against (a)biotic stresses, and JA is known to act as a secondary messenger, like other small molecules such as salicylic acid (SA) and ethylene (ET) (Vuong et al. 2014). However, to the best of our knowledge, the effects of other JA derivatives, i.e. the JA precursors OPDA and COR, a mimicking compounds of JA-Ile, the active form of JA, in plant cell culture elicitation has not been investigated so far. Therefore, another aim of the present work was to compare the effects of OPDA and COR with those of JA and MeJA on the production of stilbenes in our *V. vinifera* cv. Negramaro cell cultures.

**Figure 5:**
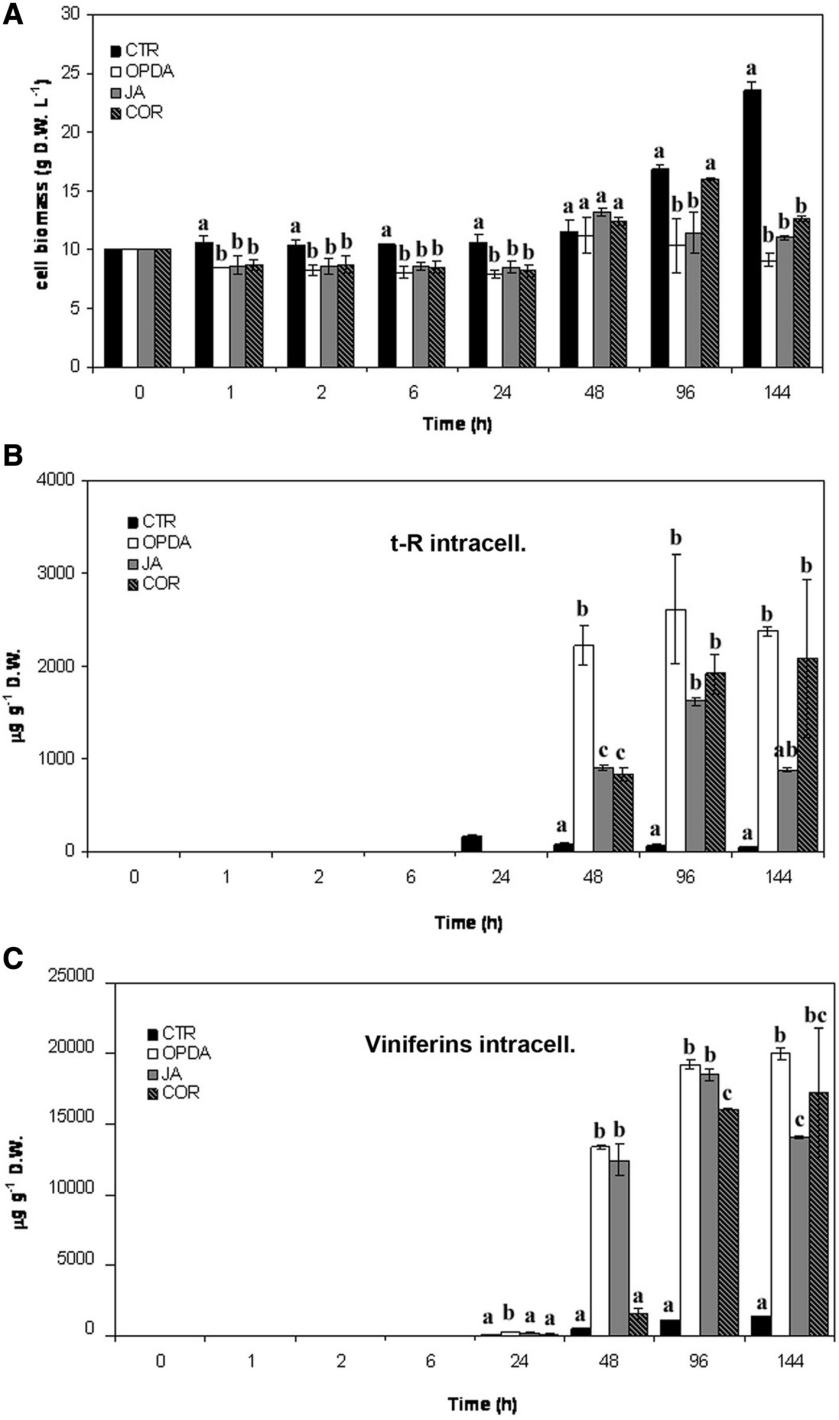
**Stilbene production in** ***V. vinifera*** **cv. Negramaro cell cultures after elicitation with OPDA, JA, and COR.** Determination of cell biomass of *V. vinifera* cv Negramaro cultures elicited with 100 μM OPDA, 100 μM JA, and 10 μM COR at different time points **(A)**. Quantification of *trans*-resveratrol (*t*-R, **B**) and viniferins **(C)** produced by Negramaro dried cell treated with different elicitors. Quantification was carried out by RP-HPLC using chemically synthesised *t*-R as the external standard. Each point is the average of three independent experiments performed in triplicate. Within any given quantification, bars with different letters indicate significant differences between different treatments (p < 0.05, ANOVA test).

In these experiments, JA and OPDA were used at the same concentration as that already used for MeJA. COR was used at 10 μM, since it was cytotoxic at higher concentrations (data not shown). As shown in Figure 5A, treatment with these three elicitors had no evident inhibitory effect on the growth of treated grape cell cultures up to 48 h. Indeed, the weight of elicited and control grape cell cultures was stable up to 24 h and started to slightly increase at 48 h. Afterwards, cell growth decreased in elicited cultures, showing a 50% reduction at 144 h in elicited cells with OPDA. The only exception was cultures elicited with COR, whose biomass increased up to 96 h at levels similar to those of control cultures.

The quantification of *t*-R with HPLC indicated that OPDA, JA, and COR, like MeJA, were able to trigger the biosynthesis of this phytoalexin. JA and COR showed a similar accumulation trend, with an increase at 48 h and a peak at 96 h. *t*-R accumulation was at about 1600 and 1800 μg g^−1^ DW (corresponding to 16 mg L^−1^ and 35 mg L^−1^) for JA and COR, respectively. Afterwards, the production dropped in cultures elicited with JA, whereas it remained sustained in those elicited with COR. OPDA elicitation resulted in an accumulation trend different from that of the other jasmonates, with a steady increase at 48 h, with about 2200 mg^−1^ DW (corresponding to 24.2 mg L^−1^), a further limited increase at 96 h, to about 2.5 mg g^−1^ DW (corresponding to 25 mg L^−1^), and sustained levels up to 144 h (Figure 5B). When all the elicitors were compared, the best performance in terms of *t*-R production was obtained with MeJA (Figure 4B), whereas about 40, 50, and 60% lower levels were recorded when the cultures were elicited with OPDA, COR, and JA, respectively.

It is noteworthy that OPDA, JA, and COR did not trigger the synthesis of *t-*P in our experimental conditions at all the time points considered here. Conversely, treatment with these elicitors resulted in intracellular accumulation of different forms of viniferins, namely *trans*-ε-viniferin, *cis*-ε-viniferin, and *trans*-δ-viniferin, as confirmed by ESI-MS analyses (see Figure 3). Chemical quantification of total viniferins (Figure 5C) indicated that *trans*-δ-viniferin was the most abundant form elicited in *V. vinifera* cv. Negramaro cell cultures, with an accumulation trend resembling that of *t-*R. The highest levels of viniferins were recorded with OPDA and JA, which were able to trigger a yield of about 18 mg g^−1^ DW (corresponding at about 200 mg L^−1^) at 96 h. The addition of COR determines similar levels of viniferins accumulation (17 mg g^−1^ DW, corresponding to about 220 mg L^−1^) at 96 and 144 h. No release of viniferins was found in the culture medium. Our results are in agreement with other authors (Santamaria et al. 2012) and suggest that specific elicitors can induce a rapid intracellular conversion of *t*-R into its dimer forms.

Taken together, these results point to jasmonates eliciting different sets of stilbenes in *V. vinifera* cv. Negramaro cell cultures. Indeed, MeJA was able to trigger high levels of *t*-R, whereas OPDA, JA, and COR elicited lower levels of this compound but remarkably high levels of viniferins and, in particular, *trans*-ε-viniferin.

To date, just a few studies have reported viniferin production in cell cultures after elicitation. Recently, δ- and ε-viniferins were identified in *V. vinifera* cv. Chasselas x *Vitis berlandieri* (Donnez et al. 2011) and *V. vinifera* cvs. Red Globe, Palieri, and Italia (Santamaria et al. 2010, 2011) cell cultures elicited with MeJA. However, viniferins were not always quantified (Donnez et al. 2011) or their amounts were much lower, about 1.45 mg g^−1^ (Santamaria et al. 2011, 2012) than those observed in this study after elicitation with OPDA, JA and COR (Figure 5C). The results reported here point to a modulatory effect of closely-related elicitor molecules on stilbene biosynthesis. Surprisingly, *V. vinifera* cv. Negramaro cell culture elicited with JA, OPDA, or COR accumulated larger amounts of viniferin than that elicited with MeJA (Figure 4B). Synthesis of these resveratrol derivatives in the cell cultures is quite interesting. Our results suggest that Negramaro cell cultures elicited with OPDA or COR could be used as an interesting model system to study viniferin biosynthesis and its physiological roles. A recent study showed that *V. vinifera* cv. ‘Pinot Noir’ cell cultures accumulated low levels of resveratrol and its glycoside *t*-P after biotic stress signals. Conversely, *V. rupestris* cell cultures, characterised by higher resistance to biotic stresses, produced massive amounts of *t*-R and the toxic δ-viniferin, indicating that resveratrol metabolism could play an important role in *Vitis* resistance (Chang et al. 2011). It was also reported that the biosynthesis of ε-viniferin in grape leaves seemed to be constitutive or inducible by biotic stress in leaves (Donnez et al. 2009; Hatmi et al. 2014). Our findings suggest that ε-viniferin was the main isoform in our elicited cell culture, confirming the physiological role of this metabolite in the response to biotic stresses mediated by jasmonates (Figures 2F and 5C).

In conclusion, *V. vinifera* cv. Negramaro grape cell cultures are a promising model system with which to study polyphenol pathway regulation in response to (a)biotic stress and biotechnological production of stilbenes. The elicitation with MeJA resulted in an intracellular accumulation of 4 mg g^−1^ (56 mg L^−1^) of *t*-R. For the first time OPDA, JA and COR were tested on *V. vinifera* cell cultures as elicitors. Remarkable amounts of intracellular viniferins (18–16 mg g^−1^, corresponding to 200–210 mg L^−1^) are produced as the result of *V. vinifera* cv. Negramaro grape cell cultures elicitation with OPDA, JA, and COR. Our findings indicate that OPDA, JA and COR molecules are powerful elicitors of this phytochemical and confirm their potential in the biotechnological production of secondary plant metabolites.

## Additional files

Additional file 1: Figure S1. Microscopic observation of *V. vinifera* cv Negramaro cell suspension, 96 h from subculture, without elicitation (CTR; panels a, d, and g) or after elicitation with 100 μM MeJA (panels b, e, and h) or 150 μM MeJA (panels c, f, and i). The images were obtained by confocal laser scanning microscopy with transmission light (panels a, b, and c), UV light after incubation with FDA to determine cell viability (panels d, e, and f), and merged images (panels g, h, and i). Scale bar: 20 μm. Additional file 2: Figure S2. Quantification of *trans*-resveratrol (*t*-R, A) and *trans*-piceid (*t*-P, B) produced by *V. vinifera* cv Negramaro cell suspension cultures treated with different concentrations of MeJA (50, 100, and 150 μM) at different time points. Quantification was carried out by RP-HPLC using chemically synthesised compounds as external standards. Each point is the average of three independent experiments performed in triplicate.

## Abbreviations

MeJA: Methyl jasmonate
CHI: Chitosan
JA: Jasmonic acid
COR: Coronatine
*t*-R: *Trans-*resveratrol
*t*-P: *trans*-piceid
OPDA: 12-oxo-phytodienoic acid
