# Supplementary figures and images for: Jasmonates elicit different sets of stilbenes in Vitis vinifera cv. Negramaro cell cultures
Source: Springerplus. 2015 Feb 1;4:49. doi: 10.1186/s40064-015-0831-z (PMC4320690; doi:10.1186/s40064-015-0831-z)

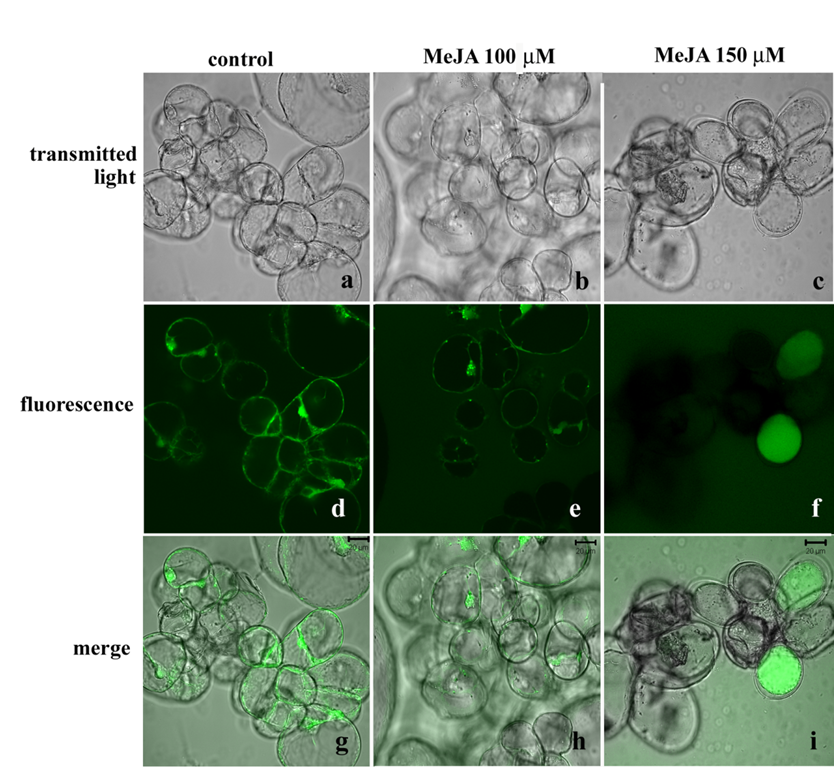

Supplement: Additional file 1: Figure S1. — Microscopic observation of V. vinifera cv Negramaro cell suspension, 96 h from subculture, without elicitation (CTR; panels a, d, and g) or after elicitation with 100 μM MeJA (panels b, e, and h) or 150 μM MeJA (panels c, f, and i). The images were obtained by confocal laser scanning microscopy with transmission light (panels a, b, and c), UV light after incubation with FDA to determine cell viability (panels d, e, and f), and merged images (panels g, h, and i). Scale bar: 20 μm. [file 40064_2015_831_MOESM1_ESM.tiff]

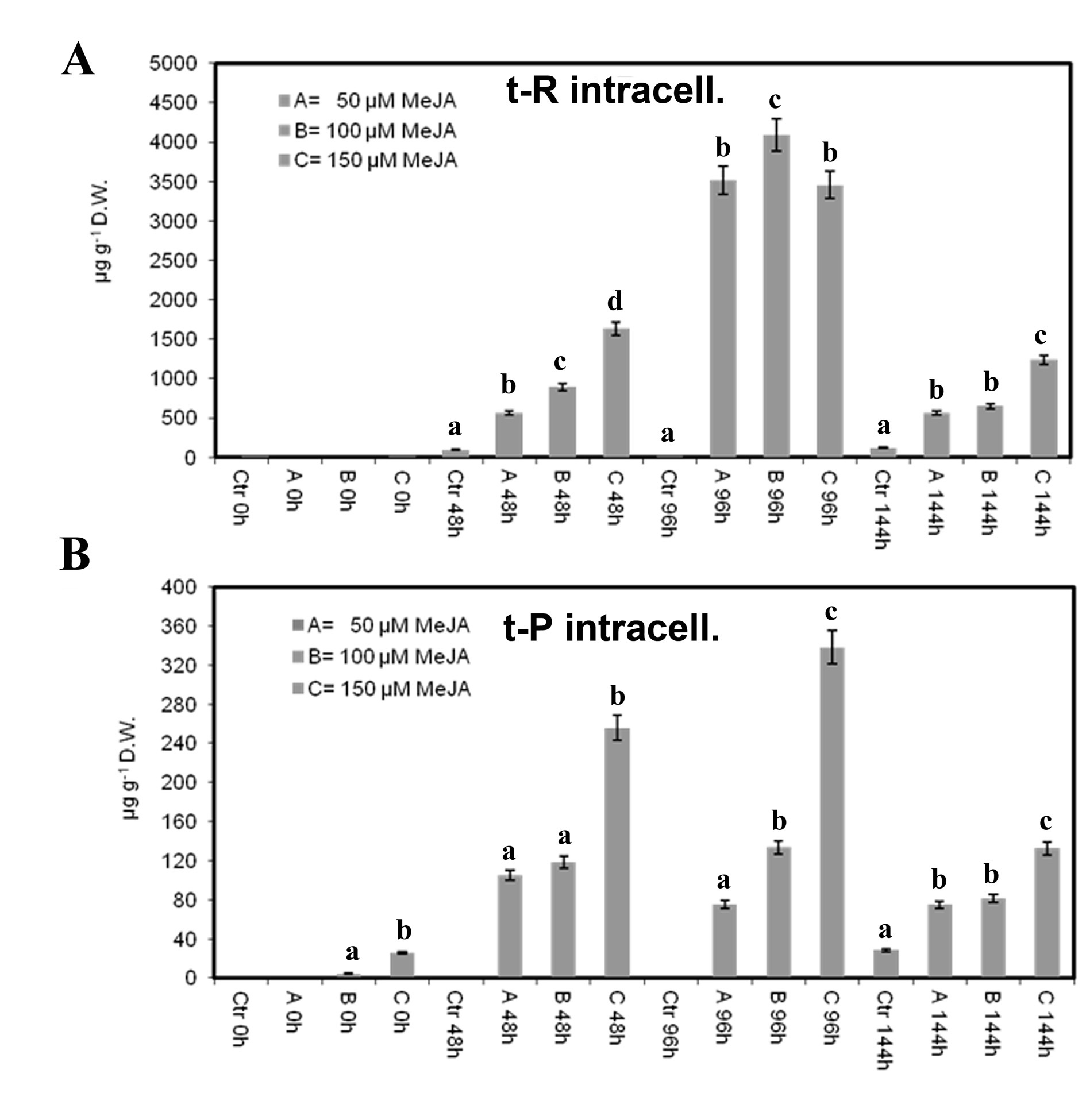

Supplement: Additional file 2: Figure S2. — Quantification of trans-resveratrol (t-R, A) and trans-piceid (t-P, B) produced by V. vinifera cv Negramaro cell suspension cultures treated with different concentrations of MeJA (50, 100, and 150 μM) at different time points. Quantification was carried out by RP-HPLC using chemically synthesised compounds as external standards. Each point is the average of three independent experiments performed in triplicate. [file 40064_2015_831_MOESM2_ESM.jpeg]
